# Clinical and biochemical spectrum of metabolic cardiomyopathy in Egyptian children

**DOI:** 10.4314/ahs.v22i1.26

**Published:** 2022-03

**Authors:** Zeinab Salah Seliem, Dina Ahmed Mehaney, Laila Abd elmoteleb Selim, Sonia Ali El-Saiedi, Reem Ibrahim Ismail, Nihal Magdi Almenabawy, Rasha Ibrahim Ammar, Inas AbdElsattar Saad, Mohammed Mosad Soliman, Mohamed A Elmonem

**Affiliations:** 1 Pediatrics Department, Pediatric cardiology division, Faculty of Medicine, Cairo University, Cairo 11562 Egypt; 2 Clinical and Chemical Pathology Department, Faculty of Medicine, Cairo University, Cairo, 11562 Egypt; 3 Pediatrics Department, Pediatric Neurology and metabolic division, Faculty of Medicine, Cairo University, Cairo 11562 Egypt

**Keywords:** Cardiomyopathy, inborn errors of metabolism, pediatric

## Abstract

**Background:**

Inborn errors of metabolism (IEMs) commonly present with pediatric cardiomyopathy. Identification of the underlying cause is necessary as it may lead to improved outcomes.

**Objectives:**

We aimed to investigate the diagnostic rate, the clinical, and biochemical spectra of IEMs among Egyptian pediatric patients presenting with cardiomyopathy, and their outcome measures.

**Methods:**

We retrospectively analyzed the clinical, biochemical, and radiological data of 1512 children diagnosed with cardiomyopathy at Cairo University Children's Hospital over a 5-year duration.

**Results:**

Two hundred twenty-nine children were clinically suspected as IEMs and underwent metabolic workup. Nineteen different IEMs were confirmed in 57 (24.4%) of the suspected children. Their median age at presentation was 2.6 years and the majority had extra-cardiac manifestations. Hypertrophic cardiomyopathy represented 43/57 (75.4%) of confirmed cases, while dilated cardiomyopathy represented 13/57 (22.8%), and one patient presented with a mixed phenotype. Twenty- six patients (45.6%) survived, while 31 patients (54%) either died or were lost to follow up and assumed deceased.

**Conclusions:**

We developed for the first time a database and a diagnostic scheme for metabolic cardiomyopathies in Egyptian children. With the recent introduction of enzyme replacement therapy, many metabolic disorders became treatable, thus establishing an early and accurate diagnosis is extremely important.

## Introduction

Cardiomyopathy (CM) is a common problem encountered by pediatricians and constitutes an important cause of morbidity and mortality in the pediatric population^1^. According to the European Society of Cardiology (ESC) classification, five types of cardiomyopathies are recognized according to the morphofunctional phenotype (hypertrophic, dilated, arrhythmogenic, restrictive, and unclassified). Each of these phenotypes can be further classified as either familial or non-familial, and whether or not the heart is the only target of the disease^2^,^3^. The etiology of pediatric CM is diverse posing a formidable diagnostic challenge. Identification of the underlying cause is of particular interest to clinicians as it may lead to improved outcomes with disease-specific treatment.

Inborn errors of metabolism (IEMs) are commonly associated with different forms of pediatric cardiomyopathies. They account for 5% of pediatric CM and about 15% of those with known causes^4^. IEMs involving CM include fatty acid oxidation defects, organic acidemias, aminoacidopathies, glycogen storage diseases, congenital defects of glycosylation, mitochondrial disorders, and lysosomal storage disorders^5^,^6^. Most IEMs are generally associated with a specific functional type of CM, which helps to limit the differential diagnosis^7^. Many IEMs are currently treatable and in many cases, the associated CM can be reversed^4^.

Due to the common practice of consanguineous marriage in the Egyptian population^8^, autosomal recessive disorders including many IEMs are much more prevalent^8^. A recent pilot study for the neonatal screening for IEMs disorders detectable by tandem mass spectrometry performed in healthy Egyptian neonates revealed a total birth prevalence of 1: 1,944, which is extremely high compared to most countries^9^. Unfortunately, epidemiological and clinical data concerning pediatric CM and its metabolic causes in Egypt, or generally in the Middle East, are extremely rare. In the current study, we retrospectively analyzed the clinical, biochemical, radiological and, neurophysiological data of all pediatric patients with the confirmed diagnosis of metabolic CM among all children presenting with CM to Cairo University Children's Hospitals (CUCH). We further discussed the outcomes of our CM patients confirmed with an IEM disorder.

## Subjects and methods

The current study retrospectively analyzed the clinical, biochemical, and radiological data of 1,512 children diagnosed with cardiomyopathy at CUCH over a 5-year duration (January 2013- December 2017). Patients' data were gathered from the databases of the cardiomyopathy and metabolic clinics, CUCH. Clinical records included relevant history details, salient clinical features, and basic laboratory data including complete blood picture, electrolytes, blood gases, liver function test, kidney function tests, creatine kinase activity, and serum lactate. Multiple radiological investigations such as echocardiography, skeletal survey, and brain MRI were reviewed. Neurophysiological tests such as electroencephalogram, electromyogram, auditory brain stem response, and electroretinogram were also reviewed.

Children with cardiomyopathy were referred for metabolic investigations if they had any of the following criteria:

1. Extra-cardiac manifestations mainly associated with neurological or developmental delay.
2. Losing motor or developmental milestones after gaining them.
3. Routine laboratory investigations suggestive of metabolic aetiology, such as metabolic acidosis, hypoglycemia, elevated transaminases, and neutropenia.
4. Strongly suggestive family history, such as previously diagnosed cases with inborn errors in the family, previous sibling death due to similar condition, and positive consanguinity.

**Figure 1:**
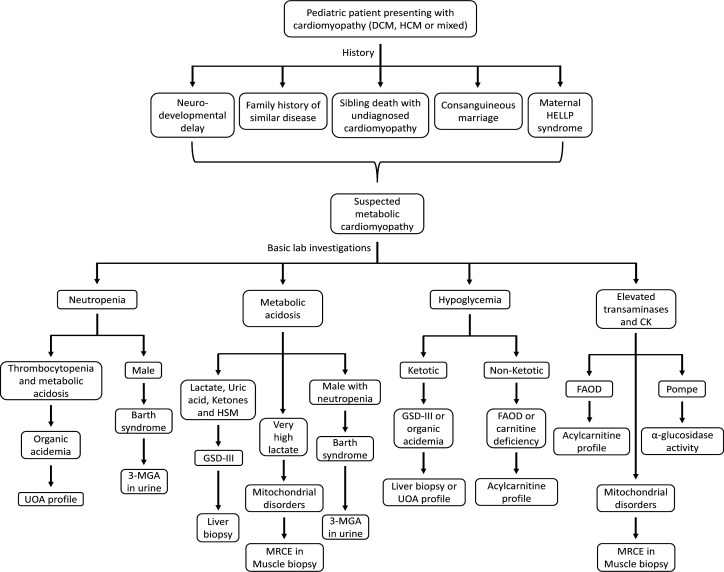
Diagrammatic scheme of the diagnostic approach to cardiomyopathy pediatric patients suspected to have metabolic etiology. CK, creatine kinase; DCM, dilated cardiomyopathy; FAOD, fatty acid oxidation disorder; GSD, glycogen storage disorder; HCM, hypertrophic cardiomyopathy; HELLP syndrome, hemoptysis-elevated liver enzymes-low platelet count associated pregnancy; HSM, hepatosplenomegaly; 3-MGA, 3-methylglutaconic acid; MRCE, mitochondrial respiratory chain enzymes; UOA, urinary organic acids. The diagnostic scheme was loosely adapted from Cox, 2007 (10).

A single criterion or a combination of them was used for the referral. Figure 1 provides a simplified scheme for the diagnosis of metabolic CM at CUCH.

According to clinical suspicion, a wide panel of metabolic investigations was performed. Enzymatic assays in patients with suspected lysosomal storage disorders, such as Pompe, Tay-Sachs, and GM1-gangliosidosis were performed in either separated leucocytes or lymphocytes using standard fluorometric techniques with the 4-methylumbelliferone based fluorophore substrates^11^,^12^. Spectrophotometric evaluation of urinary glycosaminoglycans (GAGs) by the dimethyl-methylene blue method was performed for patients suspected to have mucopolysaccharidoses followed by unidimensional electrophoretic separation to identify the types of the elevated urinary GAGs and the specific enzyme assay in isolated blood leucocytes^11^. Expanded metabolic screening (acylcarnitines and aminoacids profiling) by tandem mass spectrometry (MS/MS) using dried blood spots and urinary organic acid testing by gas chromatography/mass spectrometry (GC/MS) were performed for those suspected to have fatty acid oxidation defects, aminoacidopathies or organic acidemias^13^–^15^. Spectrophotometric assays of the respiratory chain enzymes (Complex I, II, III, and IV) in muscle homogenates were conducted in patients suspected of having mitochondrial disorders^16^. Most of the laboratory investigations were conducted at the inherited metabolic disease laboratory, Centre of Social and Preventive Medicine, Cairo University Children's Hospital.

All investigations involving human participants were in accordance with ethical standards of the institutional and national research ethics committees and with the 1964 Helsinki Declaration and its later amendments. The study was approved by the corresponding ethical committee at Cairo University Children's Hospital. All investigations performed were solely for diagnostic purposes, hence written informed consents were not required.

Quantitative data were represented as median and range, and differences between groups were conducted using the Mann-Whitney U test unless otherwise stated. Categorical comparisons between proportions were conducted using Fisher's exact test. P values were considered significant if <0.05. The stepwise Cox-proportional hazard model was performed by the SPSS software (version 16), while the Kaplan-Meier curve was created using the WinPepi statistical software package (version 11.65).

## Results

Over a 5-year period, 1,512 Egyptian children presented to the cardiomyopathy clinic at CUCH with different types of cardiomyopathies. Based on clinical history, examination, and initial imaging and laboratory investigations, 229 pediatric patients (15.2%) were suspected of having a metabolic based etiology. These children were referred for the metabolic diagnostic workup and their data were further explored in the current study.

**Figure 2:**
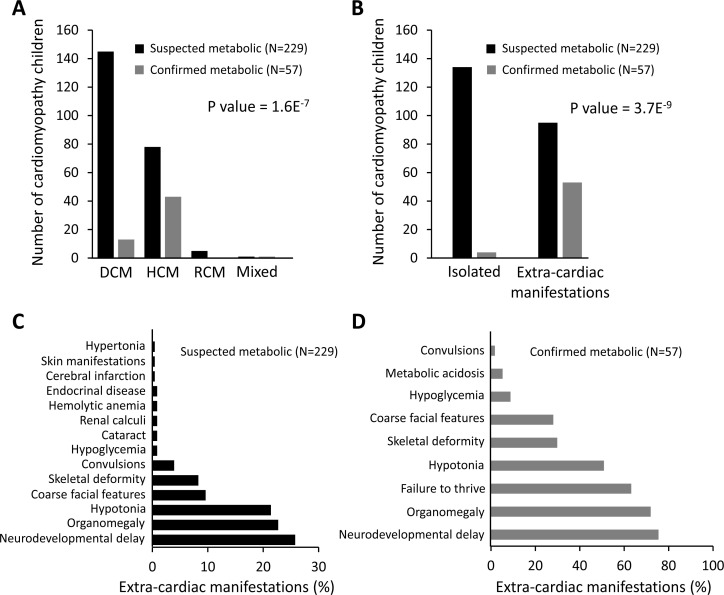
A) Distribution of cardiomyopathy morphofunctional types in children suspected or confirmed with metabolic disorders. B) Distribution of isolated cardiomyopathy and cardiomyopathy associated with extracardiac manifestations in children suspected or confirmed with metabolic disorders. P values represent nominal categorical comparisons between the confirmed and suspected metabolic cardiomyopathy groups regarding the cardiomyopathy phenotype (Figure 2A) and the incidence of extracardiac manifestations (Figure 2B) using the Fisher's exact test. C) Percentages of different associated extra-cardiac manifestations in children suspected to have a metabolic disorder. D) Percentages of different associated extra-cardiac manifestations in children confirmed with a metabolic disorder. DCM, dilated cardiomyopathy; HCM, hypertrophic cardiomyopathy; RCM, restrictive cardiomyopathy.

For the 229 suspected patients, the median (range) age at presentation was 1.8 years (1 month – 15 years). They were 123 (53.7%) males and 106 (46.3%) females. The mean duration of illness from the start of symptoms until presenting to CUCH was 11 months. Families with consanguineous parents (n=116) constituted 50.6% of the study population, while 42 patients had a previously affected sibling (18.2%). Among all suspected children, 145 (63.3%) were diagnosed with dilated cardiomyopathy (DCM), while 78 (34.1%) with hypertrophic cardiomyopathy (HCM). Restrictive cardiomyopathy (RCM) was reported in five patients (2.1%) and only one patient (0.43%) was diagnosed with mixed CM (Figure 2A). Among the suspected patients, 134 children (58.5%) presented with isolated CM, while 95 children (41.5%) had extra-cardiac manifestations (Figure 2B).

The most common extra-cardiac manifestations detected were developmental delay (59/229) (25.7%) and organomegaly (52/229) (22.7%) (Figure 2C).

Over the study duration, 57/229 (24.4%) were confirmed with 19 different IEMs. Table 1 presents the demographic data and disease distribution of the confirmed metabolic cases. Confirmed metabolic cases were 36/57 males (63.2%) and 21/57 (36.8%) females (P=0.234 compared to the suspected group). The median (range) age at presentation was a bit similar to the age of the suspected group 2.6 years (2 months – 15 years); however, the duration of illness before presenting to the cardiomyopathy clinic at CUCH was longer (20 months on average). Families with consanguineous parents (n=33) constituted 57.8% of families with confirmed metabolic cases, while 14 patients had a previously affected sibling (24.5%) and 12 patients had a history of sibling death (21%). Twenty-four children with confirmed metabolic etiology (42%) were complicated with heart failure at the time of diagnosis (Supplementary Table 1).

**Table 1:** Demographic data of Egyptian children with metabolic cardiomyopathy (N=57)

Disease group	Disease	Number of patients (%)	Mean age at diagnosis	Gender (M/F)	Positive Consanguinity	Affected siblings
**Glycogen storage** **diseases (GSD)**		**22 (38.6%)**	**3.6 years**	**15/7**	**15 (68%)**	**8 (36%)**
	Infantile Pompe (GSD-II)	16 (28.1%)	5 months	12/4	11 (68%)	8 (50%)
	Late onset Pompe (GSD-II)	2 (3.5%)	4.5 years	0/2	1 (50%)	0
	Cori (GSD-III)	4 (7%)	5.8 years	3/1	3 (75%)	0
**Mucopolysaccharidoses** **(MPS)**		**15 (26.3%)**	**4.8 years**	**9/6**	**5 (33%)**	**0**
	Hurler-Scheie (MPS-I)	5 (8.8%)	3.8 years	1/4	2 (40%)	0
	Hunter (MPS-II)	2 (3.5%)	6 years	2/0	0	0
	Morquio (MPS-IV)	3 (5.3%)	5.4 years	3/0	1 (33%)	0
	Maroteaux-Lamy (MPS-VI)	2 (3.5%)	4 years	0/2	2 (100%)	0
	MPS unspecified	3 (5.3%)		3/0		
**Mitochondrial** **disorders**		**6 (10.5%)**	**4.2 years**	**6/0**	**5 (83%)**	**2 (33%)**
	Complex I deficiency	4 (7%)	4.8 years	4/0	4 (100%)	1 (25%)
	Complex IV deficiency	1 (1.8%)	2 years	1/0	1 (100%)	1 (100%)
	Combined complex (I,II,III,IV) deficiency	1 (1.8%)	6 years	1/0	0	0
**Fatty acid oxidation** **defects (FAOD)**		**6 (10.5%)**	**2.9 years**	**2/4**	**2 (33%)**	**0**
	Primary carnitine deficiency	4 (7%)	4 years	1/3	1 (25%)	0
	LCHAD	1 (1.8%)	2.5 years	0/1	0	0
	VLCAD	1 (1.8%)	1 month	1/0	1 (100%)	0
**Aminoacidopathies**		**2 (3.5%)**	**4 months**	**1/1**	**1 (50%)**	**1 (50%)**
	Tyrosinemia type I	2 (3.5%)	4 months	1/1	1 (50%)	1 (50%)
**Lipid storage disorders**		**2 (3.5%)**	**1 year**	**2/0**	**0**	**1 (50%)**
	Tay-Sachs	1 (1.8%)	1.2 years	1/0	0	0
	Gangliosidosis M1	1 (1.8%)	9 months	1/0	0	1 (100%)
**Organic acidemias** **(OA)**		**2 (3.5%)**	**9 months**	**1/1**	**1 (50%)**	**1 (50%)**
	Methyl malonic acidemia (MMA)	1 (1.8%)	6 months	0/1	1 (100%)	1 (100%)
	Barth syndrome	1 (1.8%)	1 year	1/0	0	0
**Miscellaneous diseases**	Chanarin-Dorfman syndrome	1 (1.8%)	6 months	0/1	1 (100%)	0
	Molybdenum co-factor deficiency	1 (1.8%)	9 months	0/1	1 (100%)	1 (100%)
**Total**		**57 (100%)**	**2.8 years**	**36/21**	**33 (58%)**	**14 (25%)**

LCHAD, long chain acyl CoA dehydrogenase deficiency; VLCAD, very long chain acyl CoA dehydrogenase deficiency

Among patients with metabolic CM, 43 (75.4%) had HCM, 13 (22.8%) had DCM, while only 1(1.8%) was diagnosed with mixed CM (figure 2A). Only 4 out of 57 children (7%) had isolated CM (Figure 2B). They were all diagnosed as having primary carnitine deficiency with DCM and they were associated with varying degrees of heart failure. Most children with IEMs (93%) presented with extra-cardiac manifestations. The most common extra-cardiac manifestation was neurodevelopmental delay, which was reported in 75.4%, followed by organomegaly (71.9%) and failure to thrive (63.2%) (Figure 2D). We further compared the most important clinical features between the IEM diagnosed cases (n=57) and the IEM suspected but not confirmed cases (n=172) (Supplementary Table 2).

**Figure 3:**
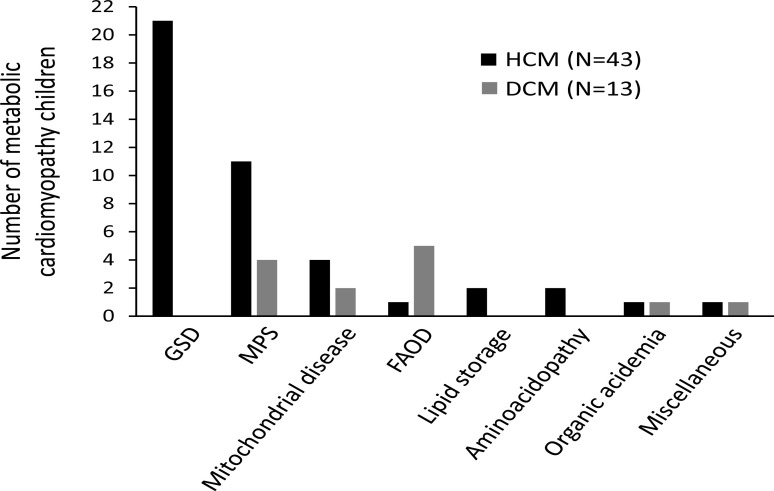
Representation of the numbers of hypertrophic and dilated cardiomyopathy patients among different groups of diagnosed metabolic disorders. DCM, dilated cardiomyopathy; FAOD, fatty acid oxidation disorders; GSD, glycogen storage disorders; HCM, hypertrophic cardiomyopathy; MPS, mucopolysaccharidoses

The most common group of IEMs causing metabolic CM was glycogen storage diseases (22 patients, 38.6%), followed by mucopolysaccharidoses (15 patients, 26.3%), mitochondrial disorders (6 patients, 10.5%), and fatty acid oxidation disorders (6 patients, 10.5%) (Table 1). Glycogen storage diseases were the most commonly diagnosed group in metabolic HCM, while fatty acid oxidation defects were the most commonly diagnosed group in metabolic DCM. Figure 3 represents the distribution of patients with different IEMs on the two major phenotypes of CM; HCM and DCM. Table 2 summarizes the most important clinical features and outcomes of the IEMs diagnosed in our cohort. Over the 5-year duration of the study, one male patient with infantile-onset Pompe disease received specific enzyme replacement therapy (alglucosidase alfa (Myozyme®), 20 mg/kg administered every 2 weeks) for 4 months but with unfavorable outcome. He succumbed to his illness at the age of 9 months due to heart failure. Another patient with late-onset Pompe disease is currently receiving enzyme replacement therapy (Myozyme®, 10 mg/kg every 2 weeks) and showing partial improvement. She was a 6 years old female patient when presented to the metabolic clinic with the main complaint of proximal muscle weakness and delayed motor development as she walked after 2 years. Her chest x-ray revealed cardiomegaly and echocardiography showed the presence of moderate hypertrophic cardiomyopathy. Her muscle biopsy demonstrated the accumulation of glycogen in myocytes. α-glucosidase enzyme assay was performed in blood lymphocytes and was deficient. After eight months of receiving ERT she showed improvement in both respratory functions (vital capacity and forced vital capacity) and motor functions (6-min walk test).

**Table 2:** Clinical data of Egyptian children with metabolic cardiomyopathy

Disease	Number of patients	Cardiomyopathy type (HCM/DCM)	Heart failure	Developmental delay	Mental subnormality	HSM	Hypotonia	Myopathy	Elevated CK	Coarse facies	Skeletal deformity	Metabolic acidosis	Hypoglycemia	Survival (alive/dead)	Other clinical features
Infantile Pompe (GSD-II)	16	16/0	16 (100%)	+++	-	+	+++	+++	+++	-	-	-	-	0/16	Death during 1^st^ year
Late onset Pompe (GSD-II)	2	2/0	0	++	+	-	++	++	+++	-	-	-	-	1/1	Respiratory problems
Cori (GSD-III)	4	4/0	0	+	-	+++	+	+	+	-	-	-	++	4/0	Hyperlipidemia
Hurler-Scheie (MPS-I)	5	3/2	1 (20%)	++	++	++	-	-	-	+++	+++	-	-	5/0	Adenoids, macrocephaly
Hunter (MPS-II)	2	1/1	0	++	++	++	-	-	-	+++	++	-	-	1/1	Spasticity, sleep apnea
Morquio (MPS-IV)	3	3/0	1 (33%)	+	-	++	-	-	-	+	+++	-	-	3/0	Only motor delay, valvular regurgitation
Maroteaux-Lamy (MPS-VI)	2	1/1	0	++	-	++	-	-	-	++	++	-	-	2/0	Corneal clouding
Complex I deficiency	4	4/0	0	++	+++	-	+++	+	++	-	-	+	-	NK	Global brain atrophy
Complex IV deficiency	1	0/1	0	++	+++	-	+++	++	+++	-	-	++	-	0/1	Leukoencephalopathy, sensory neural hearing loss
Combined complex (I,II,III,IV) deficiency	1	0/1	0	++	+++	-	+++	++	+++	-	-	-	-	0/1	
Primary carnitine deficiency	4	0/4	4 (100%)	++	-	-	+	++	-	-	-	-	+	4/0	Encephalopathy
LCHAD	1	0/1	0	-	-	++	+	+	-	-	-	-	++	0/1	Lethargy
VLCAD	1	1/0	1 (100%)	++	+	++	+	+	+	-	-	++	++	NK	
Tyrosinemia type I	2	2/0	0	+	+	+++	+	-	-	-	-	-	-	NK	Rickets, succinyl acetone in urine
Tay-Sachs	1	1/0	0	+++	+	-	++	+	-	-	-	-	-	0/1	Seizures, hypertonia, macular
Gangliosidosis M1	1	1/0	0	++	++	++	+	-	-	+	-	-	-	0/1	Poor vision, macular cherry red spots, macrocephaly
Methyl malonic acidemia (MMA)	1	1/0	0	+++	++	++	+++	-	-	-	-	+	-	1/0	Anemia, dehydration
Barth syndrome	1	0/1	1 (100%)	++	++	-	++	-	-	-	-	-	-	1/0	Neutropenia, 3-MGA in urine
Chanarin-Dorfman syndrome	1	0/1	0	++	++	++	++	++	+	-	-	-	-	1/0	Icthyosis, patchy myopathy, portal fibrosis, steatosis
Molybdenum co-factor deficiency	1	1/0	0	+++	+	-	+++	-	-	+	-	-	-	0/1	Microcephaly, seizures

+, mild; ++, moderate; +++, severe; 3-MGA, 3-methylglutaconic acid; GSD, glycogen storage disorder; LCHAD, long chain acyl CoA dehydrogenase deficiency; MPS, mucopolysaccharidoses; VLCAD, very long chain acyl CoA dehydrogenase deficiency.

Patients with primary carnitine deficiency (N=4) are currently receiving L-carnitine supplementation (100 mg/kg/day) and showing excellent response to therapy with a reversal of CM. Although they presented with isolated dilated cardiomyopathy, they were referred for further investigations since three of them had a strongly suggestive family history (one with a previously diagnosed sibling and two with previous sibling deaths and positive consanguinity), while the fourth was presenting to the clinic with significantly low free carnitine (C0) on the expanded metabolic screening by tandem mass spectrometry. He was confirmed at our laboratory and started carnitine therapy shortly after.

**Figure 4:**
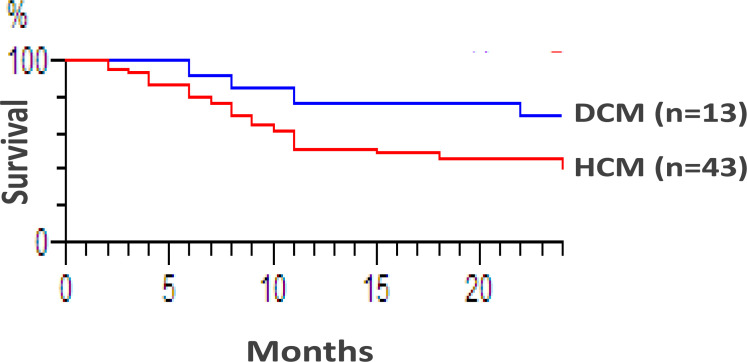
Kaplan-Meier curve for the survival rates in dilated cardiomyopathy (DCM) and hypertrophic cardiomyopathy (HCM) patients of confirmed metabolic origin.

At the end of the 5 years of the study, 26 patients (45.6%) were still alive, while 31 patients (54%) either died (24 patients) or were lost to follow up and assumed deceased (7 patients). The main cause of death in the majority of patients was heart failure mainly complicating Pompe disease (Table 2). Patients who died tended to have an earlier age at presentation (average 1.1 years) compared to still-living children (average 4.4 years). Overall mortality rate in female IEM patients (9/21, 43%) during the study period tended to be lower than male patients (22/36, 61%); however, the difference was not statistically significant (P=0.27). We further estimated the 12-month survival rate after diagnosis in the metabolic group and found that most patients presenting before one year of age died within 12 months of diagnosis. MPS patients had the best 12-month survival rate (93%) and patients with DCM had better survival rates (77%), compared to those with HCM (51%); however, the difference was not statistically significant (P=0.16) (Figure 4). By a statistical analysis using the stepwise Cox proportional hazard model, age at presentation independently affected survival in metabolic patients, as children presenting during the first year of life were 49 times more prone to die within 12 months of diagnosis (95% CI: 10.6–227.2).

## Discussion

In the current study, we reported the epidemiological data, clinical manifestations, and disease outcome of pediatric patients with metabolic CM presenting to CUCH over a 5-year period.

The median ages of the confirmed and the suspected metabolic groups were 2.6 and 1.8 years, respectively with a mild male predominance (63.2% in the 57 confirmed cases versus 53.7% in the 229 suspected cases). In a previous study carried by Cox et al. (2006) which included 916 patients suffering from different types of CM^4^, they reported that 58.2% were males and that the median age at diagnosis was 2.3 years. Male predominance might be associated with X-linked genetic disorders. The two best-known causes of X-linked CM in the current study were Hunter disease (MPS-II) and Barth syndrome.

Recurrent attacks of vomiting, lethargy, hypoglycemia or metabolic acidosis in the context of poor feeding and general bad health are important indicators of the potential presence of IEMs^4^. Multisystem involvement, delayed milestones, hypotonia, failure to thrive, coarse facial features, macroglossia, feeding difficulties, and macro or microcephaly should also warrant referral to a metabolic specialist^4^,^17^. In the current study, most children with IEMs (93%) presented with extra-cardiac manifestations, mainly neurodevelopmental delay, organomegaly, and failure to thrive.

In our suspected cohort, 63.3% of patients suffered from DCM, while 34.1% from HCM. In contrast, in the confirmed metabolic patients, HCM represented over 75% of cases which may be attributed to the abundance of GSD patients in the metabolic group, as they are strictly associated with HCM. Over half of the suspected cases with HCM (55%) were confirmed with metabolic etiology. This should raise the clinical suspicion of a metabolic disorder among HCM pediatric patients, especially if they are associated with extra-cardiac manifestations. As for DCM, metabolic disorders accounted for only a minority of suspected cases (8.9%) due to the presence of other causes of DCM among Egyptian children mainly myocarditis and familial isolated CM. Nevertheless, DCM patients with a suspicious metabolic presentation, even children with isolated CM have to undergo the metabolic workup, especially the acylcarnitines profile as the CM of such disorders could be reversed with drugs such as L-carnitine^17^.

Among the 172 patients who were not confirmed with an IEM, 45 patients reached a diagnosis (26%). Myocarditis was the most common diagnosis in this group; 25/172 patients (14.5%). Patients diagnosed with myocarditis were identified based on the combination of elevated specific cardiac biomarkers in blood, such as Troponin and CK-MB, sinus tachycardia disproportionate to the age of the patient during an electrocardiogram (ECG), and echocardiographic findings of fractional shortening and dilated left ventricle or both ventricles^18^. Histopathological confirmation was also performed in few cases of myocarditis. Another 11/172 patients (6.4%) were provisionally diagnosed as familial isolated cardiomyopathy mostly dilated although one patient had a restrictive isolated phenotype. Suspected cases with familial isolated cardiomyopathy had a strong family history. We further screened their asymptomatic relatives, mainly first-degree relatives for cardiomyopathy using ECG and echocardiography. At least two affected relatives were needed to establish the diagnosis in the index child^19^. However, since exome sequencing or genetic panel confirmation was not performed, the causative genes for familial isolated cardiomyopathies were not determined.

Colan et al. studied the epidemiology and the cause-specific outcome in pediatric HCM^20^. In accordance with our findings, they reported that IEM patients have the earliest and highest mortality rates compared to other etiologies and that patients presenting before one year of age had the broadest spectrum of causes and the poorest outcome. In the current study, Pompe disease (GSD-II) had the poorest prognosis and the highest incidence of a CM causing metabolic disorder in Egypt. It is foreseen that in the near future the prognosis of the disease can be greatly improved with the wide-spread use of enzyme replacement therapy. This would necessitate the earliest possible age at diagnosis for timely and efficient treatment. Unlike Colan et al. (2007), who reported a better survival for DCM cases compared to HCM^20^, this wasn't the case in our study probably due to our relatively limited sample size (43 HCM versus 13 DCM).

One of the limitations of our study is lacking data concerning the molecular diagnosis of metabolic patients. Determining the pathogenic mutations in the corresponding gene of each metabolic disease is essential for explaining the phenotype. It is also important for the sake of genetic counselling to prevent the occurrence of future similar cases in the family, thus minimizing the overall incidence of each disease and the metabolic CM category in general. Only one of our patients had a definitive genetic diagnosis, which is the molybdenum co-factor deficiency patient. He was confirmed to have a homozygous nonsense mutation in the MOCS1 gene (c.253C>T, p.Q85X)^21^. We are currently establishing the genetic diagnosis of both Pompe and MPS-I in our lab (being relatively common in the Egyptian pediatric population presenting with CM. Furthermore, the current database developed for this study can be the nucleus of a national registry for metabolic cardiomyopathies in Egyptian children and the basis for health care policy planning for this group of diseases in Egypt.

## Conclusion

IEMs are an important and potentially treatable group of CM in the pediatric population. Despite the diagnostic challenges posed to pediatricians, pediatric cardiologists, and laboratory geneticists, every effort should be performed to determine the underlying etiological factor in children presenting with CM. Reaching the proper diagnosis will allow for a properly timed therapeutic intervention, proper genetic counselling, and carrier detection, which would minimize the devastating outcomes and, in some diseases, may even reverse the cardiac pathology.
